# Targeting of FSP1 regulates iron homeostasis in drug-tolerant persister head and neck cancer cells via lipid-metabolism-driven ferroptosis

**DOI:** 10.18632/aging.205409

**Published:** 2024-01-10

**Authors:** Yang-Che Wu, Chin-Sheng Huang, Ming-Shou Hsieh, Chih-Ming Huang, Syahru Agung Setiawan, Chi-Tai Yeh, Kuang-Tai Kuo, Shao-Cheng Liu

**Affiliations:** 1 Department of Dentistry, Taipei Medical University-Shuang Ho Hospital, New Taipei City 23561, Taiwan; 2 School of Dentistry, College of Oral Medicine, Taipei Medical University, Taipei City 11031, Taiwan; 3 Department of Otolaryngology, Taitung Mackay Memorial Hospital, Taitung City 950408, Taiwan; 4 Department of Nursing, Tajen University, Yanpu 90741, Pingtung County, Taiwan; 5 International Ph.D. Program in Medicine, College of Medicine, Taipei Medical University, Taipei City 11031, Taiwan; 6 Department of Medical Research and Education, Taipei Medical University-Shuang Ho Hospital, New Taipei City 23561, Taiwan; 7 Continuing Education Program of Food Biotechnology Applications, College of Science and Engineering, National Taitung University, Taitung City 95092, Taiwan; 8 Division of Thoracic Surgery, Department of Surgery, School of Medicine, College of Medicine, Taipei Medical University, Taipei City 11031, Taiwan; 9 Division of Thoracic Surgery, Department of Surgery, Taipei Medical University-Shuang Ho Hospital, New Taipei City 23561, Taiwan; 10 Department of Otolaryngology-Head and Neck Surgery, Tri-Service General Hospital, National Defense Medical Center, Taipei City 114, Taiwan

**Keywords:** drug-tolerant persister cancer cells, tumor organoids, ferroptosis, FSP1

## Abstract

Background: Research has demonstrated that some tumor cells can transform into drug-tolerant persisters (DTPs), which serve as a reservoir for the recurrence of the disease. The persister state in cancer cells arises due to temporary molecular reprogramming, and exploring the genetic composition and microenvironment during the development of head and neck squamous cell carcinoma (HNSCC) can enhance our comprehension of the types of cell death that HNSCC, thus identifying potential targets for innovative therapies. This project investigated lipid-metabolism-driven ferroptosis and its role in drug resistance and DTP generation in HNSCC.

Methods: High levels of FSP1 were discovered in the tissues of patients who experienced relapse after cisplatin treatment. RNA sequencing indicated that a series of genes related to lipid metabolism were also highly expressed in tissues from these patients. Consistent results were obtained in primary DTP cells isolated from patients who experienced relapse. The Cancer Genome Atlas database confirmed this finding. This revealed that the activation of drug resistance in cancer cells is influenced by FSP1, intracellular iron homeostasis, and lipid metabolism. The regulatory roles of ferroptosis suppressor protein 1 (FSP1) in HNSCC metabolic regulation were investigated.

Results: We generated human oral squamous cell carcinoma DTP cells (HNSCC cell line) to cisplatin and observed higher expression of FSP1 and lipid-metabolism-related targets *in vitro*. The shFSP1 blockade attenuated HNSCC-DTP cell stemness and downregulated tumor invasion and the metastatic rate. We found that cisplatin induced FSP1/ACSL4 axis expression in HNSC-DTPC cells. Finally, we evaluated the HNSCC CSC-inhibitory functions of iFSP1 (a metabolic drug and ferroptosis inducer) used for neo-adjuvant chemotherapy; this was achieved by inducing ferroptosis in a patient-derived xenograft mouse model.

Conclusions: The present findings elucidate the link between iron homeostasis, ferroptosis, and cancer metabolism in HNSCC-DTP generation and acquisition of chemoresistance. The findings may serve as a suitable model for cancer treatment testing and prediction of precision treatment outcomes. In conclusion, this study provides clinically oriented platforms for evaluating metabolism-modulating drugs (FSP1 inhibitors) and new drug candidates of drug resistance and ferroptotic biomarkers.

## INTRODUCTION

Head and neck squamous cell carcinoma (HNSCC) rank as the sixth most common cancer type in terms of global incidence. HNSCC is typically treated through surgical resection combined with adjuvant radio-chemotherapy. Local recurrence frequently leads to death in HNSCC patients. Drawing inspiration from the intriguing phenomenon of bacteria resisting antibiotics, the concept of Drug-Tolerant Persister Cells (DTPCs) in cancer has emerged. These cells may enable evasion from treatment, ultimately resulting in disease recurrence, treatment resistance, and cancer progression. Non-genetic alterations, such as epigenetic modifications, play a crucial role in the development of DTPCs. These cells may be the root cause of drug resistance and recurrence in cancer patients, as they are continuously generated through metabolic reorganization mechanisms within cancer cells. Treatment strategies for head and neck squamous cell carcinoma (HNSCC), such as radiotherapy and surgical techniques, have improved considerably, and chemotherapy or monoclonal antibody use has benefitted treatments; nonetheless, more than half of treated patients with HNSCC experience disease recurrence [1]. Head and neck cancer is a major contributor to cancer mortality in men in Taiwan [2]. Treatments available for head and neck cancer include surgery, chemotherapy, and radiation therapy [3]. Early-stage head and neck cancer are typically treated with surgery, locally advanced cancer is treated with chemotherapy and radiotherapy, and recurrence or metastasis is treated with chemotherapy as a symptomatic treatment [4]. In general, even for locally advanced head and neck cancer, chemotherapy and radiotherapy are more than 50% effective [5].

However, once a tumor recurs or metastasizes or the first-line treatment fails, the effectiveness of chemotherapy and radiotherapy as a treatment decreases to below 30% [6]. Hence, treating head and neck cancer that is resistant to chemotherapy poses a significant difficulty. The epithelial–mesenchymal transition (EMT) is a key mechanism underlying cancer metastasis [7, 8]. Reportedly, the process of epithelial-to-mesenchymal transition (EMT) is linked to the development of chemotherapy resistance in cancer cells. Due to the difficulties in monitoring the transient and reversible EMT phenotype *in vivo*, the involvement of epithelial-mesenchymal transition (EMT) in metastasis has been a topic of ongoing debate. Previous studies suggest that EMT is not a prerequisite for lung metastasis, but it does lead to resistance to chemotherapy. In primary tumors comprising epithelial cells, only a small fraction of tumor cells undergoes EMT. However, these EMT cells have a crucial role in the development of recurrent lung metastases after chemotherapy, owing to their reduced proliferation, heightened expression of genes linked to apoptosis tolerance and chemo-resistance [9].

The abnormal reactivation of EMT has been associated with the acquisition of malignant properties by tumor cells during cancer progression and metastasis, including increased cell migration and invasion, enhanced tumor stemness, and heightened resistance to chemotherapy and immunotherapy. The intricate regulation of EMT is closely governed by a variety of intrinsic and extrinsic factors, such as various transcription factors, post-translational modifications, epigenetic changes, and regulatory mechanisms that are mediated by non-coding RNA [10, 11]. Previous research suggests that the EMT process is responsible for cancer cell metastasis. In the presence of high E-cadherin expression, cancer cells demonstrate an epithelial phenotype that is characterized by closely packed cells with limited motility. However, EMT causes a reduction in E-cadherin expression, resulting in a mesenchymal phenotype with loosely arranged and elongated cells that facilitate cell movement and migration. The presence of E-cadherin is essential for the adhesion of cancer-associated epithelial cells, and those with low E-cadherin expression demonstrate a higher tendency for migration. Transcription factors, such as the Snail and Slug zinc finger protein families, trigger the EMT process by inhibiting the cell-binding protein E-cadherin [12, 13]. Several published studies have linked EMT to various forms of malignant cancer cells. EMT is not a uniform process but manifests in different ways, many of which confer multiple mesenchymal cell characteristics on both normal and cancerous epithelial cells. While the above studies suggest that EMT may not be essential for cancer stemness and metastasis, they do confirm its role in drug resistance [14].

Research has demonstrated that some tumor cells become drug-tolerant persisters (DTPs) that act as reservoirs for disease relapse and drug resistance [15]. Persister cellular states are caused solely by transient molecular reprogramming in cancer cells [16, 17]. Investigation of the genetic background and microenvironment during the pathogenesis of HNSCC can improve our understanding of cell death types [18, 19]; this may provide targets for novel treatments. Cancer cells enter a reversible DTP state to avoid death from chemotherapy and targeted agents. A small percentage of cancer cells escape such cell death by entering a reversible slow proliferation state called per-sistent drug tolerance [20]. This DTP state enables cancer cells to survive drug therapy long enough to acquire drug resistance through other mechanisms [21]. Persistence is the primary obstacle to the cure of cancer, and an in-depth understanding of the biology of DTP cells and treatment strategies for their underlying mechanism may have considerable clinical significance [22]. Ferroptosis is a recently discovered form of programmed cell death that utilizes iron and is considered a potential targeted therapy for treating tumors. Nonetheless, it is unclear if genes associated with iron-dependent cell death have any prognostic significance in HNSCC. Platinum-based chemotherapy is effective as an adjuvant treatment for advanced HNSCC, but some patients may develop resistance, resulting in poor overall 5-year survival rates [23].

Ferroptosis suppressor protein 1 (FSP1) generates an antioxidant form of coenzyme Q10 (CoQ) that increases cancer cells’ resistance to ferroptosis. Studies indicate that iFSP1 treatment can enhance cancer cells’ susceptibility to ferroptosis. In preclinical tumor xenograft mouse models, ferroptosis-resistant H460 lung cancer cells’ growth was only inhibited by a double knockout of GPX4 and FSP1, not by a single knockout of GPX4 [24]. Therefore, targeting FSP1 is seen as a promising therapeutic strategy for clinical scenarios where ferroptosis resistance presents a significant challenge. This study investigated lipid-metabolism-driven ferroptosis and its role in resistance and DTP generation in HNSCC. We discovered that FSP1 expression influences lipid and glycolytic metabolism, resistance to apoptosis caused by chemotherapeutic agents, the EMT, invasion, and metastasis. This revealed that the activation of drug resistant characteristics of cancer cells is influenced by FSP1, intracellular iron homeostasis, and lipid metabolism.

## MATERIALS AND METHODS

### Patient selection and collection of clinical specimens

Surgical-residual tissue samples of 50 patients with cisplatin-resistant HNSCC were collected from Tri-Service General Hospital (TSGH) to research the expression of FSP1’s upstream and downstream targets. This study was approved by the Institutional Review Board of TSGH and was conducted in accordance with the recommendations of the Declaration of Helsinki for biomedical research (IRB:2-108-05-124). After informed consent was obtained, tissue samples were obtained from the TSGH tissue archive and retrospectively studied. We assessed the expression of FSP1 in a cohort of 50 individuals with head and neck squamous cell carcinoma (HNSCC), comprising 45 males and 5 females, aged between 29 to 75 years, with a median age of 52 years. The tissue specimens for this study were gathered from January 2015 to July 2020. Before treatment commenced, patients underwent comprehensive evaluations that included a thorough clinical history, a physical examination, a barium swallow X-ray, an endoscopy of the upper gastrointestinal tract, and CT scans of both the chest and abdomen. Treatment for all individuals was administered in accordance with the established protocols of TSGH and the NCCN guidelines. Tissue arrays were constructed using tissue samples from 50 participants: 25 normal and 25 recurrent HNSCC tissue samples. FSP1 expression in recurrent HNSCC was determined through immunohistochemical (IHC) staining of the tissue arrays. Antibodies against FSP1 (1:200; 20886-1-AP; Abcam, Waltham, MA USA) were used by following the standard IHC staining protocol and with a similar dilution of mouse immunoglobulin G as that of the negative control. FSP1 expression was analyzed by two independent pathologists. FSP1 immunoreactivity was calculated using the quick score (*Q* score) method (*Q* = *P* × I); the percentage distribution (P) of FSP1-stained tumor cells was scored from 0% to 100%, the intensity (I) of FSP1 expression was scored using a 4-point scale (3, strong staining; 2, moderate staining; 1, weak staining; and 0, no staining), and total scores ranged from 0 to 300. The feed-forward loop of oncogenic activities involved in the regulation of FSP1 and its clinical implications was investigated. This study also analyzed the expression of FSP1 and related genes in head and neck cancer by using datasets from The Cancer Genome Atlas (TCGA) and Gene Expression Omnibus (https://www.ncbi.nlm.nih.gov/geo/) databases. Expression Project for Oncology (expO) integrates gene expression data with longitudinal clinical annotations to elucidate human malignancies and provide critical insight into diagnostic markers, prognostic indicators, and therapeutic targets.

### Human tongue squamous carcinoma cell lines, drug and persister cell derivation

Cisplatin (99.7% purity, 15663-27-1) was purchased from Sigma-Aldrich (St. Louis, MO, USA). iFSP1 (99.84% purity, HY-136057, MCE, USA) is a potent, selective, and glutathione-independent inhibitor of FSP1 (apoptosis-inducing factor mitochondria-related 2, *AIFM2*) with an EC50 of 103 nM (drug information from the manufacturer, https://www.medchemexpress.com/ifsp1.html). Two kinds of human tongue squamous carcinoma cell lines were purchased from Merck (HSC-3, SCC 193, USA) and AcceGen Biotechnology (HSC-4, ABC-TC0420, USA). HSC3 and HSC4 cells were seeded in duplicate 24-well culture plates and allowed to reach 70% confluency. Persister cells were derived from treatment of the HNSCC cancer HSC3 and HSC4 cells with 5-μM cisplatin for a minimum of 9 days, with fresh drug administered every 3 days. For cells regrown from persister cells, cisplatin was subsequently removed from the persister cells and fresh cisplatin-free media was replaced every 2 days for 28 days; subsequently, this was performed for both the experiments involving persister and regrown cells.

### Transfection of HNSCC cells with shRNA targeting FSP1 and overexpression

Transient transfection cells were seeded into 24 wells at 2 × 10^4^ cells/well. TurboFect Transfection Reagents (Thermo Fisher Scientific, Waltham, MA, USA) were selected for transfection experiments. Lentivirus containing FSP1 short hairpin (sh) RNA was purchased from Thermo Fisher Scientific and prepared strictly in accordance with the manufacturer’s instructions. Two clones of shRNA were used to effectively silence FSP1 expression: A6 (shRNA1, clone ID: V2LHS-89195) and B10 (shRNA2, V3LHS-639151). shRNA lentivirus infection and construction were conducted in accordance with the standardized practice guidelines of our certified BSL-2 laboratory in the Integrated Laboratories for Translational Medicine, TSGH. Data of the pcDNA3.1 mammalian expression vector (Invitrogen, V79020) were used to design polymerase chain reaction (PCR) primers. The vector map and primer sequences are presented in Supplementary Figure 1. Ten micrograms of empty plasmid (pcDNA3.1 vector control plasmid DNA) or FSP1 expression plasmid (pcDNA3.1-CMV-FSP1) were used. The DNA-lipofectamine reagent complexes were maintained at room temperature for 30 min. The mixture was added to the well, and gentle mixing was achieved by rocking the plate back and forth. Reagent complexes did not need to be removed following transfection. The cells were incubated at 37°C in a CO2 incubator for 48 h. Successfully knocked-down cells were verified either through quantitative reverse transcription–polymerase chain reaction (qRT-PCR) or Western blotting.

### RNA preparations

Total RNA was isolated from samples (tumor tissues and cell lines) by using TriZol reagent (Life Technologies, Carlsbad, CA, USA) and quantified using NanoDrop (Thermo Fisher Scientific). RNA from exosomes was extracted using a miRNeasy Micro Kit (QIAGEN, Hilden, Germany). In brief, exosome suspension (20 μL) was mixed with QIAzol lysis buffer (700 μL) and processed in accordance with the vendor’s protocol. RNA samples were subsequently eluted with 25 μL of RNase-free water (Invitrogen™ 10977015, repeated twice with 25 μL of RNase-free water to concentrate the samples). The RNA concentration in the samples was again determined using NanoDrop.

### Quantitative real-time PCR (RT-qPCR)

Total RNA was extracted using a TriZol reagent (Invitrogen) in accordance with the manufacturer’s protocol. Complementary DNA (cDNA) was synthesized from 1.0 μg of total RNA by using oligo (dT) primers and the PrimeScript II First Strand cDNA Synthesis Kit (Takara, Shiga, Japan). Subsequently, a qRT-PCR kit was used to assess the expression levels of CDK4 and related targets on an MxPro real-time PCR system (Agilent Technologies, Stratagene Mx3005P, USA). GAPDH was used as the internal reference gene. The qRT-PCR reaction was performed as follows: 95°C, 10 min; 95°C, 10 s; 60°C, 20 s; 75°C, 15 s; 40 cycles. A ΔΔCt method was used to determine relative gene expression from qPCR data with GAPDH as an endogenous REF gene. Supplementary Table 1 shows the Q-PCR primer list.

### Western blotting

Western blotting was used to determine the quantities of protein and related message transfer proteins. First, 10%, 12.5%, and 15% sodium dodecyl sulfate–polyacrylamide gel electrophoresis films were prepared. These were placed in an electrophoresis tank, and electrophoresis buffer (Tris-Glycine-SDS Buffer, Sigma, T7777-1L) was added. Next, 4 μL of loading buffer was added to a 16-μL sample (total protein 20 μg) of the solution. The sample was cooled and denatured (100°C, 10 min) and then placed on the electrophoresis sheet. Electrophoresis separation was performed at 100 V. After approximately 3 h, the gel was removed, and protein transfer was performed. The gel was then placed in ice-cold transfer buffer. The gel was covered with presoaked polyvinylidene difluoride (PVDF) paper and placed in a transfer holder. After the 1-h transfer, the PVDF paper was added to the blocking buffer (CD-110500, CANDOR Bioscience, Wangen im Allgäu, Germany) and shaken for 1 h at room temperature. Primary antibody was added to the TBST buffer (BR510, Biomate, Taipei, Taiwan). The solution was allowed to react overnight at 4°C or at 37°C for 2 h, and it was washed three times with washing buffer (TBS + 0.05% Tween 20) for 10 min each time. Subsequently, the secondary antibody was added to the TBST buffer, and they were allowed to react at room temperature for 1 h and then washed with washing buffer three times for 10 min each time. Finally, the color was developed using an ECL luminescence system, and the result was quantified using a densitometer (Alphalmage 2000, Alpha Innotech, San Leandro, CA, USA). Supplementary Table 2 shows the antibody list.

### Tumor spheroid formation assay

HNSCC persister cells were transferred to serum-free low-adhesion culture plates containing Dulbecco’s modified Eagle medium/F-12 containing N2 supplement (Invitrogen), 20 ng/mL EGF, and 20 ng/mL basic fibroblast growth factor (stem-cell medium; PeproTech, Rocky Hill, NJ, USA) for 2 weeks to allow tumor sphere formation. The spheres were counted under a microscope. The tumor ball formation efficiency was calculated as the ratio of the number of balls to the number of implanted cells.

### Cell migration assays

HNSCC persister cells were seeded and cultured in six-well plates for 24 h. The cells were incubated with mitomycin (10 μg/mL) for 1 h. A linear scratch was created by moving the tip of a 200-μL pipette through the cell monolayer. Cellular debris was removed, and the cells were allowed to migrate for 24–48 h. Gap healing was determined using a microscope (Nikon, Japan) from micrographs taken before and after the wound was created. Migration distance was measured from images (three random fields) obtained at indicated time points. The gap size was subsequently analyzed using ImageJ software (Wayne Rasband National Institutes of Health, Bethesda, MD, USA).

### Cell invasion assays

An invasion assay was performed in accordance with a previously established protocol. In brief, 3 × 10^5^ HNSCC persister cells were seeded onto matrigel (BD Biosciences, San Jose, CA, USA) in culture plate inserts (pore size of 8 μm, Corning Inc., Corning, NY, USA) in serum-free medium. Three independent and random fields per well were photographed and the number of cells per field was counted. An average of the three determinations was obtained for each chamber. Each invasion assay was performed a minimum of three times.

### Fatty acid metabolism assay

The experiment described involves using the Lipid Extraction Kit (Chloroform Free, ab211044, Abcam) for analyzing cell lysates to study the interaction of FSP1 with lipid metabolism. Pellet 5 × 10^5^ cells by centrifugation at 1000 × g for 5 minutes. The cell pellet was washed once with PBS (Phosphate-Buffered Saline). The washed pellet was then resuspended in 25–50 μL of PBS. For the lipid extraction, 25 μL of the cell suspension was transferred to a clean 1.5 mL microcentrifuge tube 500 μL of Lipid Extraction Buffer which was added to the cells. This mixture was vortexed immediately for 1–2 minutes. The homogenate was then centrifuged at 10,000 × g for 5 minutes at 4°C, and the supernatant was collected. The supernatant was agitated on an orbital shaker at room temperature for 15 minutes. A subsequent centrifugation step at 10,000 × g for 5 minutes was performed, and the lipid-containing supernatant was carefully transferred to a new tube. The volume of the supernatant was recorded, and then it was dried in a vacuum concentrator or a 37°C incubator overnight until a thin film was visible, indicating complete drying. The dried lipid sample was then ready for further processing. These steps are conducted as per the instructions provided in the kit’s manual, and they are crucial for the successful extraction and analysis of lipids from cell samples. Subsequent quantitative determination was performed using Human Fatty Acid Oxidation In-Cell ELISA Kit (ab118182, Abcam) and Cholesterol/Cholesteryl Ester Assay Kit (ab65359, Abcam).

### Patient-derived xenograft mouse models

Xenografting was performed with mice homozygous for the severe combined immune deficient (SCID) mutation. Twenty-five 8-week-old female nonobese diabetic (NOD) and SCID mice obtained from BioLASCO Taiwan (Taipei, Taiwan) were bred under standard experimental pathogen-free conditions in accordance with the protocols of the Animal Care and Use Committee of Taipei Medical University (Approval number: LAC-2021-0657 and LAC-2021-0377). Briefly, primary HNSCC tumors were placed in RPMI 1640 in an ice bath in the surgical site. Thin slices of tumor were cut into 2–3 mm3 pieces and washed three times with RPMI 1640. The samples were minced into fine fragments that would pass through an 18-gauge needle. They were then mixed 1:1 (v/v) with Matrigel (Collaborative Research, Bedford, MA, USA), achieving a total volume of 0.2 mL per injection. The tissue mixture was injected subcutaneously in both flanks of the 8-week-old male SCID mice. Twenty 8-week-old female NOD and SCID mice obtained from BioLASCO Taiwan were bred under standard experimental pathogen-free conditions. The mice were divided into four groups (untreated, cisplatin, dapagliflozin, or combination therapy; five per group). The mice received different treatments: vehicle (PBS orally five times per week) or dapagliflozin (10 mg/kg, orally three times per week). Tumor volume was measured using a standard caliper every other week with the following formula:


V = (L × W2)/2

where L is the long axis and W is the width of the tumor. Animals were humanely sacrificed following the experiments, and tumor and tissue samples were collected for further analyses.

### Immunohistochemical staining

Tissues collected from the experimental animal sacrifice were fixed in 10% (vol/vol) formalin for 24 h and embedded in paraffin. Bones were decalcified prior to paraffin embedding. Paraffin-embedded 4-μm tissue sections were dewaxed through incubation with xylene for 2 min twice and were rehydrated with 100% ethanol twice for 2 min, 95% ethanol for 2 min, 75% ethanol for 2 min, and ddH_2_O for 2 min. The tissue sections were stained with hematoxylin for 2 min, washed with tap water for 10 min, counterstained with eosin for 30 s, and then dehydrated with 75% ethanol for 30 s twice, 95% ethanol for 30 s, and 100% ethanol and xylene for 30 s. After being mounted using mounting medium, the stained tissue sections were examined under a light microscope, and the tumor areas were digitally photographed. Tumor areas in the images were calculated using ImageJ software, version 1.5.

### Statistical analysis

SPSS (IBM, Armonk, NY, USA) was used to perform all statistical analyses. Each experiment was performed three times. All data in the figures are expressed as mean ± standard deviation. Comparisons between groups were performed using the *t*-test. All statistical tests were two-sided, and *p* < 0.05 was considered significant. Continuous data were analyzed using the paired *t*-test or Wilcoxon rank test. Categorical data were analyzed using χ2 or the Fisher exact test. Survival analysis was estimated using the Kaplan–Meier method with the log-rank test to calculate differences between curves.

### Availability of data and materials

The datasets that are used and analyzed by the current investigation will be provided by the corresponding author in reply to the reasonable demands. Experimental procedures, characterization of new compounds, and all other data supporting the findings are available in the supplementary materials. 

## RESULTS

### FSP1 is highly expressed in tissues procured from cisplatin-resistant patients and is correlated with FSP1 expression

**Figure 1:**
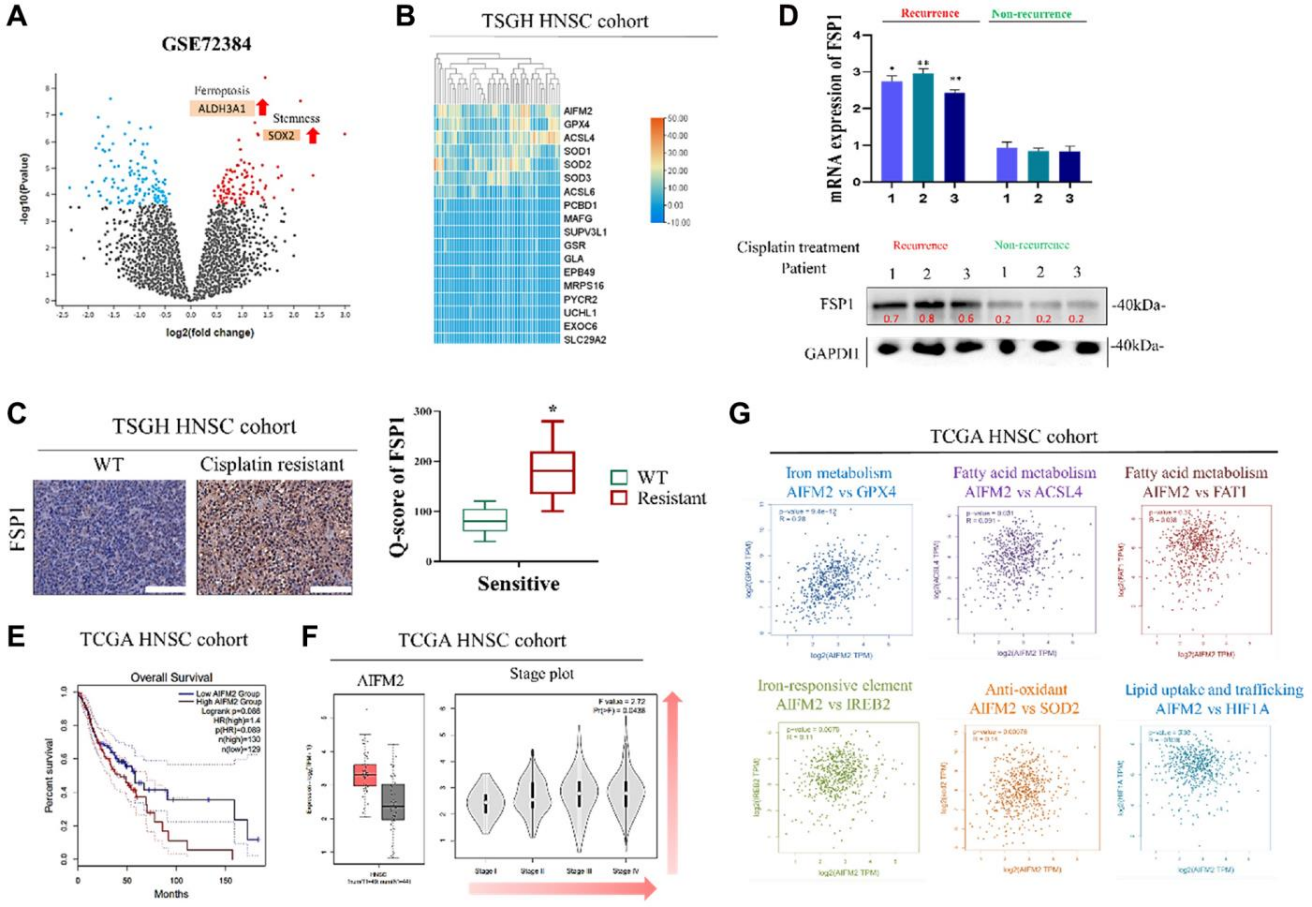
**Ferroptotic biomarker FSP1 is aberrantly expressed in human HNSCC tissues.** (**A**) Volcano Plot analysis from the Gene Expression Omnibus (GEO) database (GSE72384). (**B**) Heat map of expression of FSP1 and related genes in TSGH HNSCC cohort (*n* = 50). (**C**) IHC staining of FSP1 expression in cisplatin-treatment HNSCC recurrence tissues (left) and nonrecurrence tissues (right) and Q score of FSP1 expression in patients with HNSCC. (**D**) Graphical representation of FSP1 expression in patients with HNSCC (top image: FSP1 expression in patient tissue; bottom image: FSP1 mRNA expression in patient tissue). (**E**) Kaplan–Meier curves indicating the effects of low and high BTK expression on the overall survival of patients with HNSCC (*n* = 259). (**F**) Ferroptotic biomarkers of FSP1 were aberrantly expressed in box and stage plot of the TCGA HNSCC cohort. (**G**) Analysis of correlations of FSP1 with iron metabolism, fatty acid metabolism, Iron-responsive element and antioxidant related genes. ^*^p < 0.05, ^**^p < 0.01.

Our study focused on examining the genes that become active following cisplatin treatment in HNSCC cells that are resistant to chemotherapy. We used data from the Gene Expression Omnibus (GEO) database (GSE72384) to conduct our investigation. Through a Volcano Plot analysis, we identified two key indicators where red dots signify genes with increased expression levels in the TS samples compared to the control samples, and blue dots indicate genes with decreased expression levels. Our results showed that the gene ALDH3A1, associated with Ferroptosis biomarkers, and the gene SOX2, involved in cancer stemness, were both upregulated (Figure 1A). Related clinical tables of FSP1 expression and correlation analysis are presented in Table 1. Subsequently, we examined the gene expression profiles associated with HNSC tissue samples listed in Table 1. A gene expression heat map is displayed in Figure 1B. Gene sequencing from the cohort (provided in Table 1) indicated that FSP1 and related regulatory genes were highly expressed in patients’ tissues. In our preliminary clinical observations, the following results were obtained from the patient-derived primary cells that were subjected to tissue staining and separation. As presented in Figure 1C, FSP1 was more highly expressed in tissues procured from cisplatin-resistant patients compared with those from non-cisplatin-resistant patients, and cisplatin resistance was correlated with FSP1 expression. The IHC results showed that the expression level of FSP1 in HNSCC tumor tissues was significantly increased (*p* < 0.05). The analysis of FSP1 expression in patient tissues, both at the mRNA and protein level, revealed that samples subjected to chemotherapy displayed elevated levels of FSP1 compared to untreated samples. This suggests that the deviant FSP1 expression subsequent to chemotherapy may have a potential regulatory role in cancer cells. Consistent results were obtained for the tissues isolated from the specimens of patients with relapsed HNSCC. Regardless of whether mRNA or protein was considered, the cisplatin-treated recurrence samples exhibited higher FSP1 expression than the nonrecurrence samples (Figure 1D). To further clarify the effect of abnormal FSP1 expression in head and neck cancer on patient survival, we used online open databases to analyze and support our hypothesis. The TCGA database revealed that poorer survival of patients with higher expression of FSP1 (*AIFM2*) (Figure 1E) and that FSP1 is highly abnormally expressed in head and neck cancer tissues and expression increases with tumor progression (Figure 1F). Finally, Figure 1G illustrates the high expression of FSP1 in head and neck cancer. Significant positive correlations with GPX4 (ferroptosis), ACSL4 (fatty acid metabolism), SOD2 (antioxidant), HIF1A (tumor growth and metastasis), FAT1 (fatty acid metabolism) and IREB2 (Iron-responsive element). The outcomes of our investigation were in line with those of the wider TCGA HNSC cohort. These preliminary findings indicate that there may exist a regulatory association between FSP1 and cancer cell metabolism with regards to various aspects such as fatty acids, antioxidation, and iron ions. It suggests that FSP1 expression could potentially have a role in regulating these biological processes in cancer cells. Further research is necessary to establish the precise nature of this relationship and to explore the underlying molecular mechanisms involved.

**Table 1:** Correlation between FSP1 (AIFM2) expression and clinicopathological variables of TSGH-HNSCC patients (*n* = 50).

**Clinicopathological Variables**		**No.**	**FSP1 (*AIFM2*)**		**x^2^**	***p*-value**
			**High expression**	**Low expression**		
Age, years	≤65	25	10	15	2.885	0.089
	>65	25	16	9		
Gender	Male	45	27	18	0.000	1.000
	Female	5	3	2		
Differentiation	Well/Moderately	14	4	10	10.593	0.001
	Poor	36	28	8		
Tumor size (mm)	≤50	15	4	11	0.019	0.891
	>50	35	10	25		
Lymph node metastasis	N0	10	3	7	5.433	0.020
	N1-N2	40	28	12		
Primary stage	I+II	25	10	15	5.194805	0.022654
	III+IV	25	18	7		

### Cisplatin DTP HNSCC cancer cell line generation and validation

**Figure 2:**
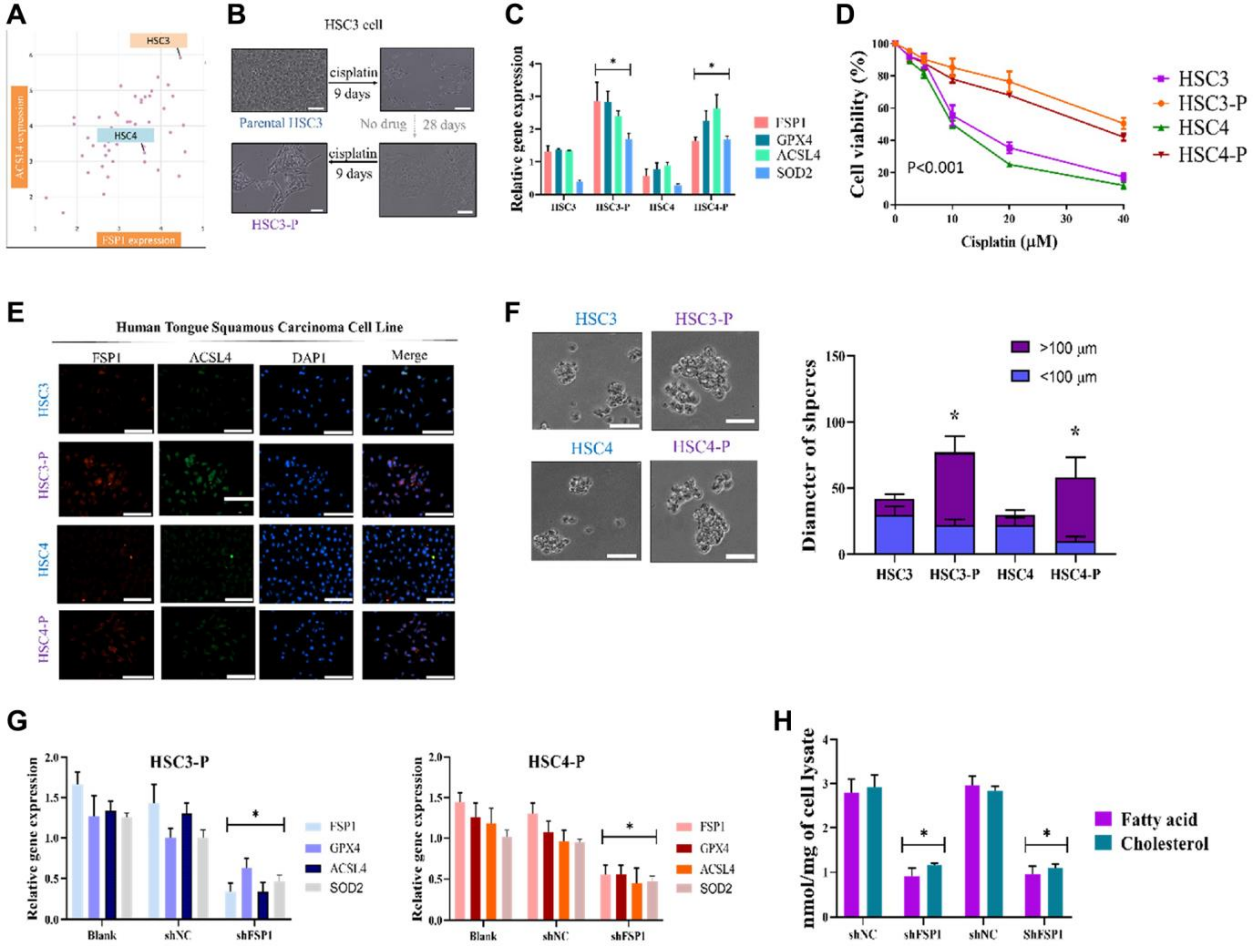
**Cisplatin DTP HNSCC cancer cell line generation and validation.** (**A**) Cell line expression analysis to optimize the experimental parameters used HSC3; HSC4 was the control. (**B**) HSC3 parental cells were treated with 5 μg/mL cisplatin for 9 days, removed from cisplatin for 28 days, and then reincubated with cisplatin for 9 days to generate a stable DTP cell line. (**C**) Relative gene expression assay indicated higher expression of FSP1, GPX4, and ACSL4 in cisplatin-DTP cell lines. (**D**) HSC3-P was compared with parental counterpart in terms of sensitivity to cisplatin treatment for validation. (**E**) Immunofluorescence staining analysis of the expression of FSP1 and ACSL4 in the DTP HNSCC cancer cell line. (**F**) Diameter of tumor spheres and (**G**) relative gene expression of FSP1, GPX4, and ACSL4 with and without shFSP1 transfection. (**H**) Downregulation of lipid components following FSP1 suppression. (^*^p < 0.05, ^**^p < 0.01, ^***^p < 0.001). Scale bar: 5 μm.

We first established human tongue squamous cell carcinoma cell lines HSC3 and HSC4 DTP cancer cells that could tolerate cisplatin. Two kinds of human tongue squamous carcinoma cell lines were purchased from Merck (HSC-3, SCC193, USA) and AcceGen Biotechnology (HSC-4, ABC-TC0420, USA). Figure 2A presents the analysis of the expression of FSP1 and ACSL4 in the HNSCC cell line. We used the depmap online tool to investigate the vulnerabilities of cancer and identify targets for therapeutic development (https://depmap.org/). The findings indicate that HSC3 and HSC4 cell lines are relevant to the expression of *AIFM2* and ACSL4 and can be utilized in this study. Out of the two cell lines, HSC3 exhibits a greater expression of *AIFM2* compared to HSC4. It is hypothesized that following Cisplatin treatment, more pronounced differences could be observed, and HSC3 will serve as a research sample for subsequent ferroptosis studies. HSC3 was used as the experimental cell line in this study, and HSC4 was used as the control cell line. Subsequently, we generated drug-tolerant persister cells from these two cell lines. HNSCC cancer cell lines were treated for approximately 9 days with a cytotoxic concentration of cisplatin (5 μM; Figure 2B). The results revealed that a small fraction of HNSCC cancer cells (3%–5%) entered a quiescent (persister) state to evade the strong selective pressure of high-concentration cisplatin (Figure 2B, upper right panel). We assessed whether these persister cells were consistent with the reported observation of a reversible state of drug resistance. Removal of cisplatin (the cells were cultured in cisplatin-free media for >28 days) allowed the persister cells to regrow (Figure 2B, lower right panel). Subsequent cisplatin treatment led to persister cells being derived again and indicated that these cells had reacquired sensitivity to cisplatin (Figure 2B, lower left panel). The reversibility of drug resistance in HNSCC cancer persister cells, which has also been reported in HSC3 persister cell models, is indicative of a resistance mechanism. Relative gene expression assay findings indicated higher expression of FSP1, GPX4, and ACSL4 in cisplatin-DTP cell lines (presented in Figure 2C). The rederived persister cells were discovered to be sensitive to cisplatin. These cells are named cisplatin-DTP cells and further annotated as HSC3-P and HSC4-P. The results indicated that the persister cells were considerably more resistant to cisplatin than were their parental cells (Figure 2D). We compared DTP and non-DTP cell line functional phenotypes. In cell immunostaining, we found abnormal expression of FSP1 and ACSL4 in both DTP cell types (Figure 2E). The DTP cell phenotypes were characterized by poor migration and high drug resistance. As previously reported, DTP HNSCC cell lines have strong stemness properties, indicated by greater diameters of formed spheres (Figure 2F). The relative gene expression survey of FSP1, GPX4, and ACSL4 with and without shFSP1 transfection is presented in Figure 2G. In an effort to provide more concrete evidence of the crucial role played by FSP1 in the regulation of lipid metabolism in head and neck squamous cell carcinoma (HNSCC) drug-tolerant persisters (DTP) cells, we conducted a detailed analysis focused on the effects of FSP1 knockdown on the metabolic pathways within these cells. Specifically, we targeted the FSP1 gene for silencing and subsequently monitored the resultant changes in lipid metabolism. The data obtained from this investigation was quite telling; it demonstrated a marked reduction in the cellular concentrations of key lipid constituents, namely fatty acids and cholesterol. These findings are critical as they suggest that FSP1 is a significant regulator of lipid metabolism in these cancer cells. The observed downregulation of lipid components following FSP1 suppression could have profound implications for the metabolic state and viability of the HNSCC DTP cells. This pivotal data is comprehensively illustrated in Figure 2H, where a clear visual representation of the reduced lipid levels post-FSP1 silencing is presented.

### Overexpression of FSP1 enhances cellular function in HSC cell lines and is reversed by FSP1 inhibitors

**Figure 3:**
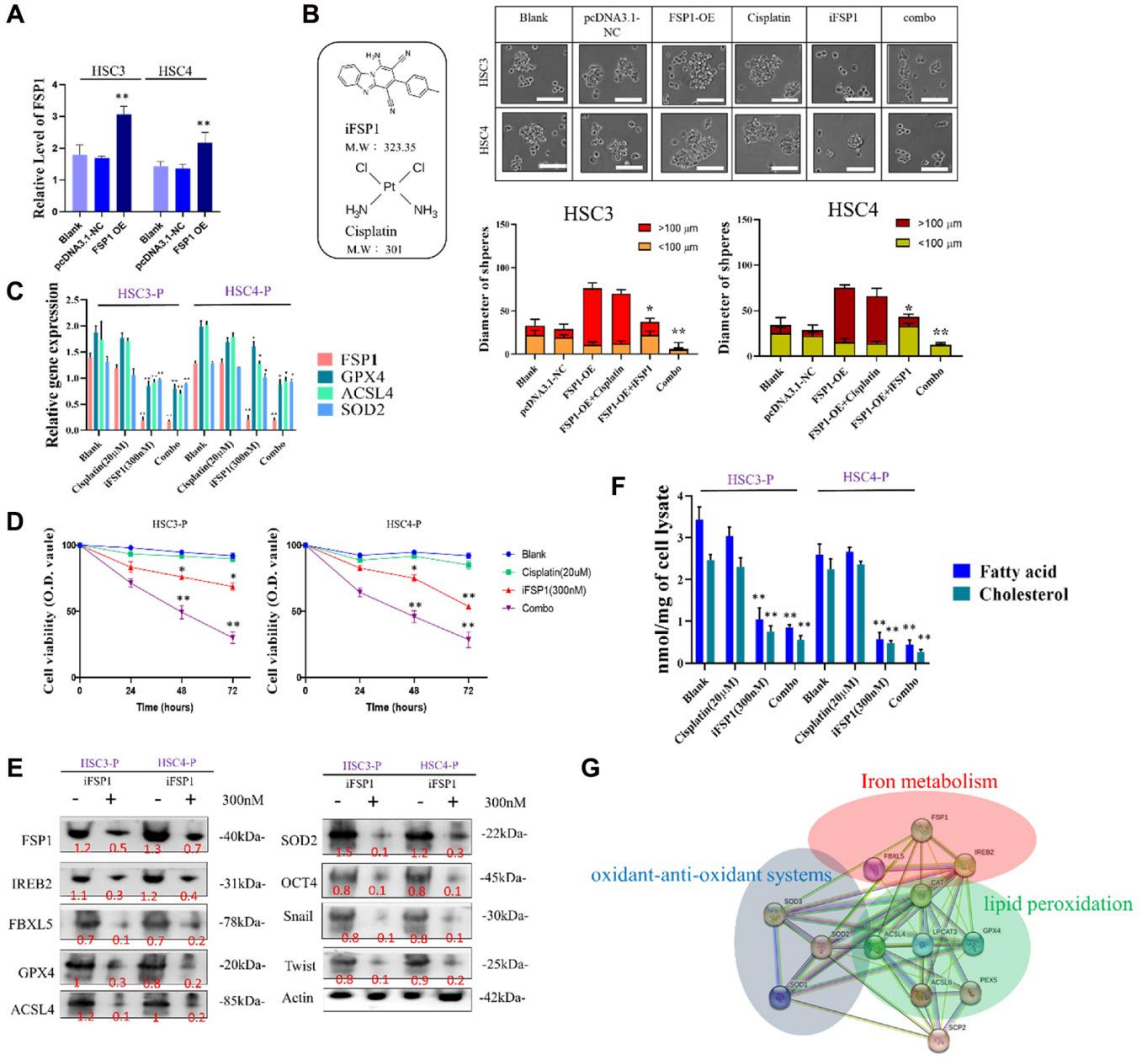
**Overexpression of FSP1 enhanced cellular function in HSC cells and was reversed by FSP1 inhibitor.** (**A**) Overexpression of FSP1 in the transfected HSC cell line. (**B**) Diameter of tumor spheres. (**C**) RNA analysis of related genes of HSC DTP cells through FSP1 inhibitor treatment. (**D**) HSC DTP cells were compared with parental counterparts in terms of their sensitivity to cisplatin, FSP1 inhibitor, and combination treatment. (**E**) Western blotting indicated the expression levels of iron-metabolism-related markers FSP1, IREB2, FBXL5, and GPX4; fatty acid metabolism marker ACSL4; and antioxidant marker SOD2 in HSC DTP cells. Actin was used as a loading control. (**F**) Downregulation of lipid components following FSP1 inhibition. (**G**) Potential regulatory network of ferroptosis in cells, involving three pathways: ferroptosis, fatty acid metabolism, and cellular antioxidation. (^*^p < 0.05, ^**^p < 0.01, ^***^p < 0.001). Scale bar: 5 μm.

To elucidate the role of FSP1 in conferring drug resistance, we performed a thorough investigation on two HSC cell lines. Using pCDNA 3.1 as our expression vector, we successfully overexpressed FSP1 in these cells, with confirmation of this overexpression demonstrated in Figure 3A. A subsequent cell migration assay provided evidence that FSP1 overexpression enhances the motility of HSC cells, an effect that was not mitigated by treatment with cisplatin. Interestingly, the introduction of an FSP1 inhibitor not only reduced cell migration but also allowed cisplatin to exhibit a more potent inhibitory effect on the migration rate, as shown in Figure 3B. Furthermore, RNA analysis of the HSC DTP cell lines, which show persistent cisplatin resistance, revealed atypical expression patterns of genes implicated in ferroptosis (FSP1), fatty acid metabolism (ACSL4), and cellular antioxidation (SOD2). This dysregulated gene expression profile was normalized upon the application of an FSP1 inhibitor, suggesting a reversal of the resistant phenotype, as depicted in Figure 3C. The alteration in gene expression correlating with cellular responses was further supported by cell viability assays, confirming these observations, as seen in Figure 3D. Delving deeper into the effects of FSP1 inhibition on the biological characteristics of HNSCC-DTP cells, including their stemness, invasive capacity, and metastatic potential, we employed immunoblotting techniques. This allowed us to dissect the downstream protein expression related to ferroptosis regulation. The findings presented in Figure 3E shed light on the potential molecular mechanisms by which FSP1 modulates these characteristics in DTP cells. To affirm FSP1’s role in lipid metabolism in HNSCC DTP cells, we scrutinized the consequences of FSP1 suppression on lipid-related cellular activities. The results unambiguously indicated that silencing FSP1 leads to a significant reduction in the intracellular concentration of fatty acids and cholesterol, as illustrated in Figure 3F. This supports the hypothesis that FSP1 is integral to the proper regulation of lipid metabolism. Lastly, Figure 3G presents a comprehensive overview of the putative regulatory network of ferroptosis within the cell, highlighting three intertwined pathways: ferroptosis itself, fatty acid metabolism, and cellular antioxidation. This diagram serves to visualize the complex interactions and regulatory mechanisms that may be affected by the perturbation of FSP1 activity within these cells, further emphasizing its pivotal role in cellular metabolism and drug resistance phenomena.

### Inhibition of FSP1 significantly suppresses metastasis in patient-derived xenograft mouse models *in vivo*

**Figure 4:**
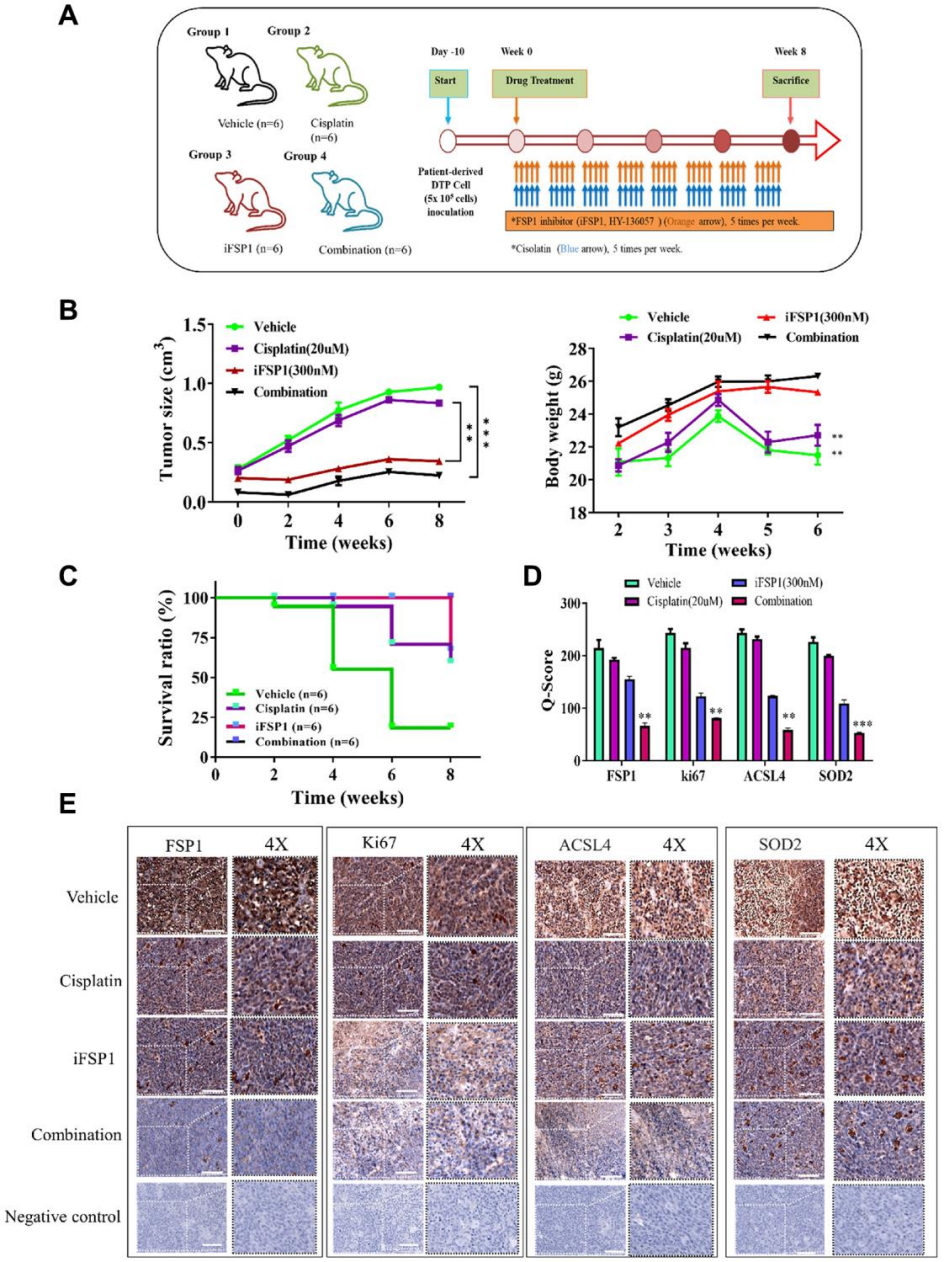
**Inhibition FSP1 significantly suppressed metastasis in the patient-derived xenograft mouse model.** (**A**) Flowchart showing the *in vivo* experimental design and treatment schedule. (**B**) Tumor size and body weight curve over time indicated that the combination of cisplatin and FSP1 inhibitor suppressed tumor growth and caused no apparent systematic toxicity. (**C**) The Kaplan–Meier survival curve indicated that the group of mice receiving combined treatment had a higher survival ratio than did the other groups. (**D**) The Q-score of tissue staining. (**E**) Immunostaining analysis of tumor sections indicated that the combined treatment prominently suppressed FSP1, ACSL4, SOD2, and ki67 expression compared with other sections. (^**^p < 0.01; ^***^p < 0.001).

We evaluated the therapeutic effects of iFSP1 by creating a patient-derived xenograft mouse model through orthotopic inoculation with patient-derived tumor cells. Patient-derived tumor cells were injected into the right flank of NOD-SCID female mice for *in vivo* validation of the findings in the *in vitro* study. The mice were divided into four groups: vehicle control, cisplatin alone (orally five times per week), iFSP1 alone (orally five times per week), and the combination of both drugs (combining both regimens), respectively. A flowchart showing the *in vivo* experimental design and treatment schedule is presented in Figure 4A. The tumors that developed in the mice receiving the combination treatment were markedly smaller at the indicated time points than those that developed in the control mice, with a 1.6-fold difference in tumor size by week 8 (*p* < 0.01). However, no significant effect on mouse bodyweight at week 6 was observed (Figure 4B). Furthermore, the mice in the combination treatment group had a considerably higher survival rate than those in the other groups (Figure 4C). Using tumor samples derived from the tumor xenograft mouse model, we demonstrated that the expression of FSP1, ki67, SOD2, and ACSL4 proteins was significantly suppressed in the FSP1 inhibition and combined treatment groups compared with that in the control group. The Q score of tissue staining was also calculated. The findings indicated that FSP1 plays a crucial role in the malignant progression of HNSCC and in the modulation of markers (Figure 4D, 4E).

## DISCUSSION

A small population of cancer cells can evade cell death induced by chemotherapy and targeted therapy by entering a reversible slow-proliferative state, which is referred to as the drug-resistant persistent (DTP) state. In this state, cancer cells can survive drug treatment long enough to develop additional resistance mechanisms. Consequently, cancer persistence is a significant obstacle in achieving a cure for cancer. A thorough comprehension of the biology of DTP cells and the development of therapeutic strategies targeting this mechanism could have significant clinical implications. DTP cells have been reported to adapt to new environments through epigenome modification, transcriptome regulation, flexible energy metabolism, and interaction with the tumor microenvironment [25–27]. Ferroptosis is a newly described cell death, which is driven by iron accumulation and lipid peroxidation. [28] Increasing evidence suggests that ferroptosis can impact antitumor immunity and may provide viable approaches to improve the response to immune checkpoint inhibitors (ICIs). Furthermore, ICIs can augment tumoral ferroptosis induced by CD8+ cells, whereas greater resistance of cancer cells to ferroptosis may reduce the efficacy of ICIs [29].

Iron-dependent cell death causes not only iron metabolism disorders but also the accumulation of numerous cell membrane lipid peroxidation products, which in turn induce cell death [30]. However, iron-dependent cell death reportedly plays a major role in the pathophysiology of cancer, neurodegenerative diseases, and damage to the brain, kidneys, liver, and other organs [31, 32]. In addition to common programmed death, apoptosis can be caused by changes in the environment. Studies have reported that iron-dependent cell death is induced when iron and oxygen are present in the body [33]. Iron-dependent cell death is caused by the accumulation of lipid superoxide free radicals (lipid reactive oxygen species) in the body when the body’s antioxidant capacity is incapable of inducing metabolic abnormalities [34]. Iron ions are present in the body in two main forms: divalent iron and trivalent iron [35]. In the process of transporting iron ions, iron continually changes from the divalent to the trivalent state. This reaction process is called the Fenton reaction [36]. The electrons and ions released in the Fenton reaction induce hydroxyl radicals. These free radicals further attack the polyunsaturated acyl chains of the phospholipid bilayer on the cell membrane and induce the generation of more lipid radicals. Stable free radicals continually attack the phospholipids (propagation phase) on the cell membrane through the release of protons, producing more unstable reactive lipid peroxyl radicals, further increasing the oxidative stress in the body [37].

Emerging research indicates that drug-tolerant persister (DTP) cells can adapt to diverse conditions by modifying their epigenome, regulating the transcriptome, altering energy metabolism, and interacting with the tumor microenvironment. Despite the discovery of the primary ferroptosis pathway involving GSH-GPX4, the precise regulatory network underlying the drug-tolerant state’s role in head and neck squamous cell carcinoma (HNSC) remains largely unclear. Moreover, directly targeting GPX4 in clinical settings is currently impractical due to the lack of a safe, proven selective inhibitor [38]. Therefore, identifying potential alternative regulatory mechanisms to target remains an unresolved issue.

Glutathione is a key antioxidant in the body; it is composed of three amino acids: cysteine, glutamate, and glycine, of which cysteine is the most important [39]. When the concentration of cysteine in the cytoplasm is sufficient, cysteine can maintain the balance of oxidative stress in the body and prevent such stress from causing damage. Glutathione hydroperoxidase 4 (GPX4) catalyzing the reduction of peroxides at the expense of reduced GSH, and selenium is present in the form of selenocysteine in the GPX4 protein [40]. When the concentration of glutathione in the body is insufficient, the activity of GPX4 is reduced and the superoxide free radicals in the body are not eliminated, which eventually induces iron-dependent cell death [40]. Ferroptosis is an iron-dependent form of necrotic cell death and is characterized by oxidative damage to phospholipids. Ferroptosis is generally considered to be controlled only by the phospholipid hydroperoxide reductase GPX4 and free-radical-trapping antioxidants [41]. However, investigations of the basic factors underlying the sensitivity of specific cell types to iron death are essential for understanding the pathophysiological effects of iron death and related applications in cancer treatment [41]. Although metabolic restriction and phospholipid components cause iron death sensitivity, a cell-autonomous mechanism explaining cells’ resistance to iron death has not been reported [42]. *AIFM2*, which is also known as iron death suppressor protein 1 (FSP1), was initially identified as a gene that induces apoptosis while providing protection against iron death triggered by GPX4 deletion. Developing a comprehensive understanding of the genetic and environmental factors involved in the development of HNSCC, which influence the sensitivity or resistance of cells to specific types of cell death, may facilitate the development of innovative treatment approaches. FSP1 expression affects altered lipid and glycolytic metabolism, resistance to apoptosis resulting from chemotherapy, epithelial-to-mesenchymal transition (EMT), invasion, and metastasis [43].

FSP1 functions as an oxidoreductase that leverages NADPH to convert CoQ into its reduced form, CoQH2, similar to how the GSH-GPX4 antioxidant system uses NADPH to replenish GSH via glutathione reductase (GR). Consequently, NADPH is crucial for the action of FSP1, facilitating the regeneration of CoQH2 to combat ferroptosis. Significantly, various enzymes that produce NADPH, such as glucose-6-phosphate dehydrogenase (G6PD), 6-phosphogluconate dehydrogenase (PGD), isocitrate dehydrogenase 1 (IDH1), and malic enzyme 1 (ME1), are targets of NRF2. This enhanced ability to produce NADPH is vital for offsetting the heightened consumption of NADPH by FSP1 during the production of CoQH2, which could also contribute to the resistance to ferroptosis observed in KEAP1 deficient lung cancer cells. This explains why these NADPH-producing enzymes are selected as targets by NRF2 and are markedly elevated in KEAP1 mutant lung cancers. Furthermore, eliminating FSP1 in A549 cells leads to an increased NADP+/NADPH ratio, while overexpressing FSP1 in H1299 cells yields the reverse effect [44]. Subsequent research revealed that the level of FSP1 expression imparts resilience against ferroptosis in lung cancer cells with mutations in or lacking KEAP1. This is evidenced by the fact that hindering FSP1, either through genetic deletion or with a pharmacological inhibitor, made KEAP1-deficient cells more vulnerable to ferroptosis. Conversely, increasing the expression of FSP1 enhanced the resistance to ferroptosis.

GPX4 is responsible for regulating ferroptosis by converting lipid hydroperoxides into harmless lipid alcohols with the assistance of glutathione. However, inhibiting GPX4 has been ineffective in inducing ferroptosis in several cancer cell lines, suggesting the existence of an alternative resistance mechanism. Through unbiased genetic screening, FSP1 has been identified as a novel ferroptosis suppressor protein and a secondary regulator of ferroptosis after GPX4. FSP1 is mainly found at the periphery of lipid droplets and the plasma membrane, with some overlapping with the endoplasmic reticulum and Golgi. Bersuker et al. and Doll et al. have both independently reported that N-myristylation of FSP1 is essential for its anti-ferroptotic function [45, 46].

Resistance to chemotherapy and radiotherapy often leads to the failure of conventional cancer treatments. Ferroptosis, a form of cell death characterized by the buildup of lipid peroxidation (LPO), is crucial in overcoming these resistances in tumors [47]. It has been noted that modulators of LPO can effectively combat cancers that are resistant to multiple drugs. Cancer cells can adapt during chemotherapy, giving rise to multi-drug resistant cells that emerge from a pool of persister cells. Targeting these persister cells, which are linked to a mesenchymal state, could help prevent cancer recurrence. Research indicates that this mesenchymal and therapy-resistant state is reliant on GPX4, an enzyme that defends against ferroptosis. For example, vemurafenib, which targets mutant BRAF, can lead to dedifferentiation in melanomas, making them more susceptible to ferroptosis, as evidenced by significant changes in lipid composition, including an increase in polyunsaturated fatty acids (PUFAs) [48]. Furthermore, studies have shown that the mesenchymal state is connected with the transcription factor ZEB1, which is a central regulator of lipid metabolism and can promote the stemness, colonization ability, and metabolic adaptability of cancer cells. This suggests a link between ferroptosis, lipid metabolism, and drug-resistant phenotypes. Ferroptosis also plays a role in determining a cancer’s sensitivity to radiotherapy. Ionizing radiation boosts both the expression of ACSL4—a key enzyme in lipid metabolism required for ferroptosis—and the accumulation of LPO, leading to ferroptosis. The removal of ACSL4 can significantly counteract ferroptosis induced by radiation, enhancing radio resistance. Moreover, the inhibition of 12-LOX can alter the radiosensitivity of human prostate cancer cells, hinting at a P53/12-LOX-mediated, ACSL4-independent pathway in the control of radio resistance. These insights into the interaction between ferroptosis and lipid metabolism offer promising avenues to increase the effectiveness of cancer treatments through radiation and chemotherapy [49].

Recent advancements have identified the CoQ10/FSP1 axis as a key defense mechanism against ferroptosis, offering a promising therapeutic target for hepatocellular carcinoma (HCC) by suppressing tumor growth through ferroptosis induction. Additionally, ferroptotic cell death may boost both innate and adaptive anti-tumor immune responses, potentially improving treatment outcomes. Flow cytometry has shown that inhibiting FSP1 significantly increases the presence of macrophages, dendritic cells, and T cells in HCC tumors, while also markedly reducing tumor size without negatively impacting body weight in animal models. The body possesses mechanisms to prevent ferroptosis, such as the GPX4 and FSP1 pathways, which inhibit excessive lipid oxidation [50]. Targeting these pathways can trigger ferroptosis, but targeting GPX4 may harm normal cells due to the absence of suitable inhibitor binding sites. FSP1, conversely, seems to be a safer target as FSP1-deficient mice develop normally and the protein has multiple drug-binding pockets. The similarity in structure between brequinar, a clinical trial drug for cancer, and the FSP1 inhibitor iFSP1 led to the hypothesis of off-target effects, which was supported when brequinar was found to inhibit FSP1 at high concentrations and fit into FSP1’s CoQ10-binding pocket. FSP1 knockout cells did not show increased ferroptosis, suggesting specific inhibitor effects are necessary [51]. A screening of around 10,000 compounds identified icFSP1, which induces ferroptosis in various cancer cells and causes FSP1 to separate from other proteins, aggregating and inducing ferroptosis. This mechanism was confirmed through fluorescence microscopy and further validated by the reduction of tumor growth in melanoma-engrafted mice treated with icFSP1. The research also delved into DHODH inhibitors, which had been known to increase ferroptosis sensitivity in cancer cells. DHODH, critical for nucleic acid synthesis, was found to inhibit ferroptosis by reducing CoQ10 in mitochondria. However, brequinar requires doses much higher than those needed for DHODH inhibition to enhance ferroptosis sensitivity, indicating potential off-target effects due to structural similarities with iFSP1 [51]. Efforts are currently focused on improving the *in vivo* stability of these drugs through chemical modifications to harness the full potential of FSP1 inhibition as a novel approach to cancer therapy distinct from other ferroptosis inducers.

In our initial study, we noted a significant effect of FSP1 in tissue samples from patients who experienced a recurrence following cisplatin therapy. RNA sequencing revealed a substantial upregulation of genes involved in lipid metabolism in these recurrent cases. These findings align with observations in primary DTP cells from relapsed patient samples. A search of the TCGA database further validated this discovery. There appears to be a connection between the emergence of drug resistance in cancer cells and the roles of FSP1, intracellular iron balance, and lipid metabolism, as shown in Figure 1. FSP1’s relationship with various metabolic facets, including fatty acid processing, antioxidative responses, and iron ion regulation, hints at its potential regulatory impact on cancer cell biology. In our experiments with human oral squamous cell carcinoma DTP cells (HNSCC cell line) exposed to cisplatin, we noted elevated levels of FSP1 and lipid metabolism markers. Suppressing FSP1 reduced the stem-like qualities of HNSCC-DTP cells and decreased their invasiveness and metastatic potential. Cisplatin treatment seemed to stimulate the FSP1/ACSL4 axis in these cells, as depicted in Figure 2. Additionally, we investigated the role of FSP1 overexpression in drug resistance across two HSC cell lines. We found that FSP1 overexpression led to increased cell migration, which cisplatin could not suppress. RNA analyses of two HSC DTP cell lines that continued to resist cisplatin showed irregular gene expression patterns related to ferroptosis (FSP1), fatty acid metabolism (ACSL4), and cellular antioxidants (SOD2). However, inhibiting FSP1 reversed these anomalies. Figure 3 displays the proposed interaction network of cell ferroptosis, incorporating pathways of ferroptosis, fatty acid metabolism, and antioxidation. Lastly, we assessed the HNSCC CSC-suppressing effects of iFSP1, a metabolic agent and ferroptosis inducer, in pre-surgical treatment, by promoting ferroptosis in a patient-derived xenograft mouse model, illustrated in Figure 4.

Targeting FSP1 is suggested as a new approach in the paradigm shift of treating HNSCC. This study is limited by the fact that cell lines and SCC-HN cancers exhibit genetic heterogeneity and are in a constant state of evolution. Moreover, even *in vivo*, daughter cells undergo frequent gains and losses of chromosomal material when an anaphase bridge forms and breaks, which occurs roughly every six to seven anaphases. This phenomenon has been demonstrated in previous studies [52]. Consequently, the efficacy of treating DNA-damage-induced drug resistance may be questionable. In the future, these drugs may not be used to support immunotherapy or, at a minimum, non-DNA damaging therapies. In addition, it is now known that just about every therapeutic approach and especially drugs like cisplatin cause senescence in the cancer environment which has been suggested to lead to side effects and may lead to relapse by secreting tumour promoters especially [53, 54]. Nonetheless, our current discoveries provide new insights into FSP1 as a fresh biomarker for ferroptosis in DTP HNSCC cells, improving our comprehension of this aspect of cancer biology.

## CONCLUSIONS

**Figure 5:**
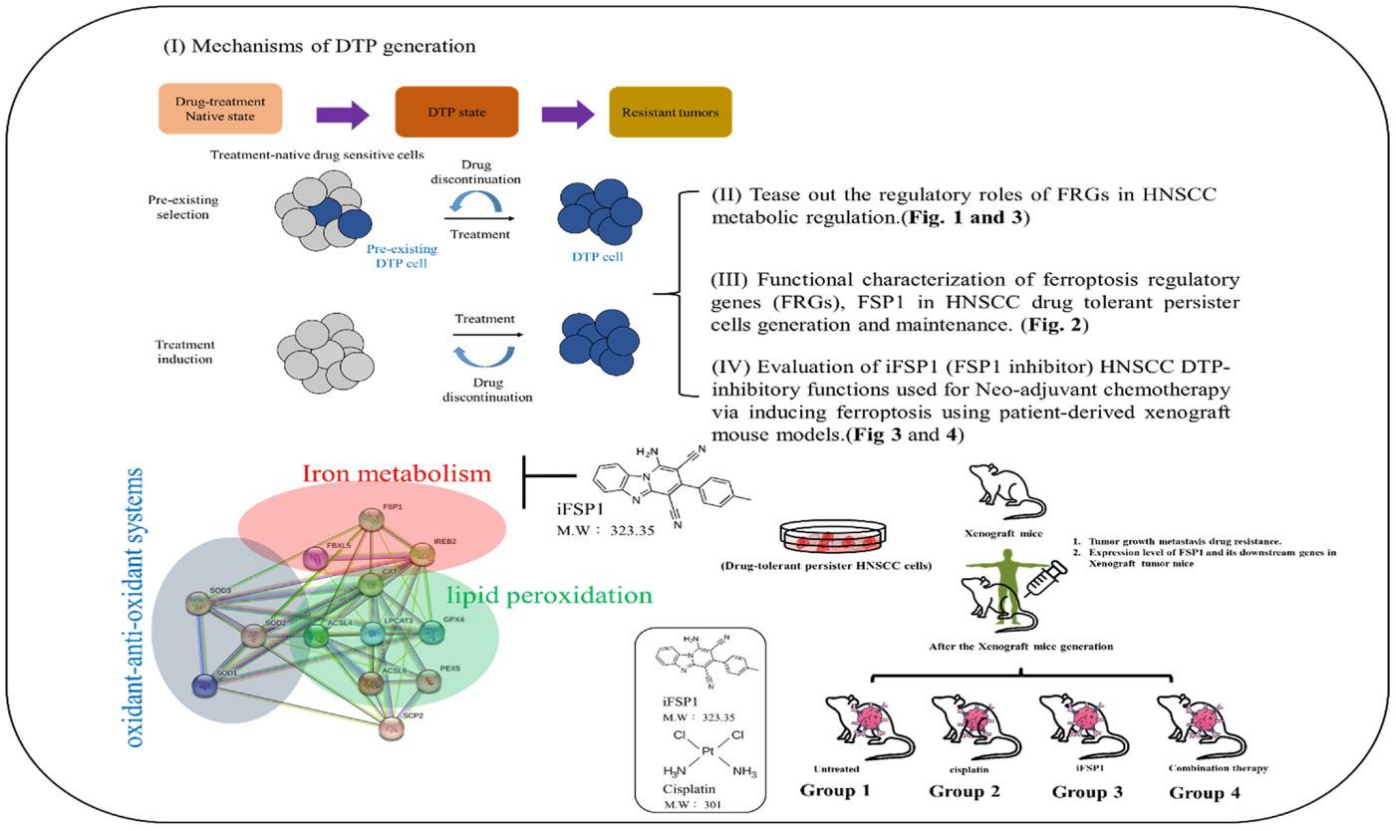
**Graphical summary of the mechanisms underlying DTP generation.** Our findings were analyzed with regard to cell viability, death, lipid reactive oxygen species, iron production, and mRNA and protein expression and interaction.

As presented in the pictorial abstract shown in Figure 5, we demonstrated that FSP1 downregulation suppressed numerous potential axis pathways leading to decreased migration, invasion, colony, and sphere formation. We explored therapeutically applicable vulnerabilities of minimal residual disease DTP cells by using an experimental model. The establishment of HNSCC-DTP cell and PDX mouse models provides clinically oriented platforms for evaluating metabolism-modulating medication. The results of this study enhance the understanding of FSP1 as a novel ferroptotic biomarker, its involvement in the process of DTP HNSCCs, and it’s *in vitro* and antitumor effects in animals. Focusing on FSP1 as a therapeutic target is a promising approach in cancer treatment, and it could also be an important biomarker for categorizing patients and tailoring individual treatments. Nonetheless, to fully comprehend how FSP1 is regulated and how it interacts with other regulators of ferroptosis, more research is required. There is a critical need to refine agents that inhibit FSP1 to improve their selectivity, effectiveness, and safety for clinical use. Extensive clinical trials are essential to establish the value of FSP1 as a biomarker for prognosis and treatment prediction. Moreover, combining FSP1 inhibitors with existing cancer treatments may amplify the overall therapeutic outcome.

## Supplementary Materials

Supplementary Figure 1

Supplementary Tables
