# Supplementary material for: Targeting of FSP1 regulates iron homeostasis in drug-tolerant persister head and neck cancer cells via lipid-metabolism-driven ferroptosis
Source: Aging (Albany NY). 2024 Jan 10;16(1):627–47. doi: 10.18632/aging.205409 (PMC10817390; doi:10.18632/aging.205409)
Supplement: Supplementary Figure 1 [file aging-16-205409-s001.pdf]

SUPPLEMENTARY FIGURE

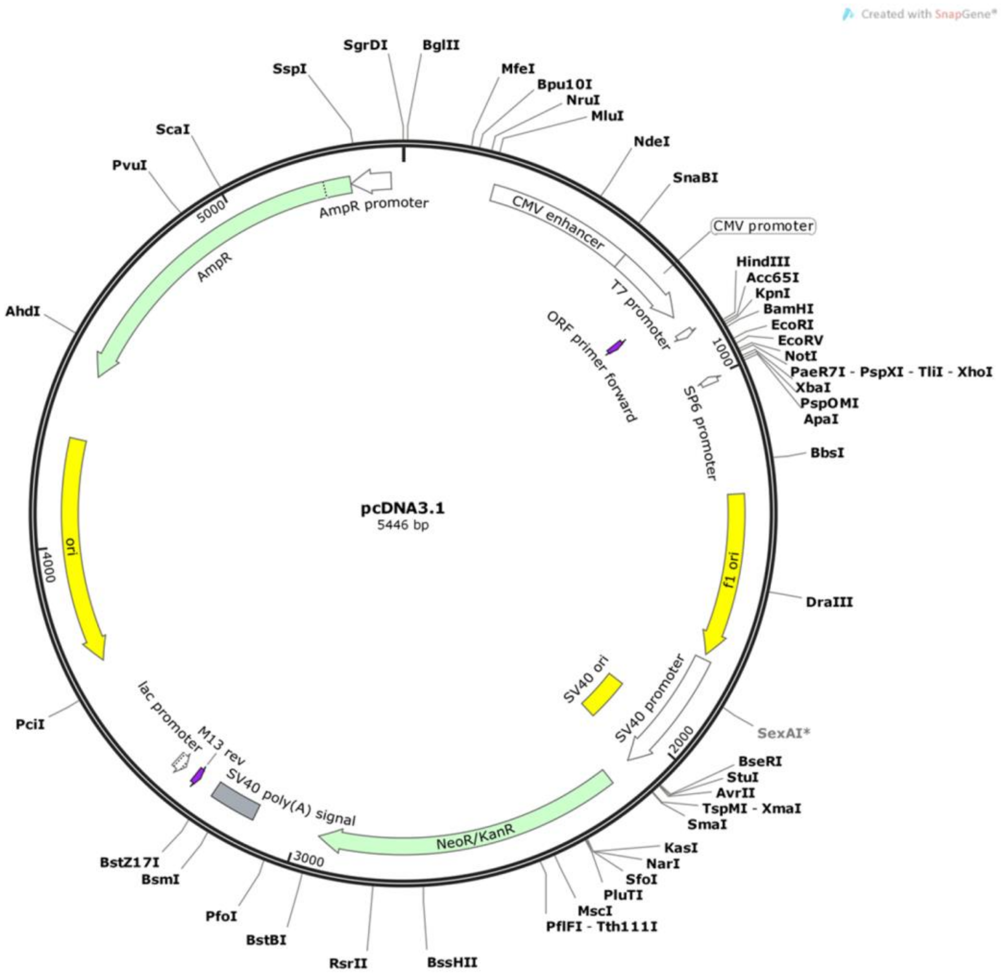

Primer	Sequence
FSP1-ECOR1-forward	AATTGAATTCTCTTGGTCTGGTCTCAACGG
FSP1-Xba1-reversed	AATTTCTAGATGTCACCCTCTTTGCCTGAG

Supplementary Figure 1. Overexpression of FSP1 plasmid backbone and primer design.
