# Supplementary material for: Targeting of FSP1 regulates iron homeostasis in drug-tolerant persister head and neck cancer cells via lipid-metabolism-driven ferroptosis
Source: Aging (Albany NY). 2024 Jan 10;16(1):627–47. doi: 10.18632/aging.205409 (PMC10817390; doi:10.18632/aging.205409)
Supplement: Supplementary Tables [file aging-16-205409-s002.pdf]

SUPPLEMENTARY TABLES

Supplementary Table 1. Q-PCR primer list.

Primer	Forward	Reverse
FSP1	TCTTGGTCTGGTCTCAACGG	TGTCACCCTCTTTGCCTGAG
GPX4	ACAAGAACGGCTGCGTGGTGAA	GCCACACACTTGTGGAGCTAGA
ACSL4	TGGAAGTCCATATCGCTCTGT	TTGGCTACAGCATGGTCAAA
SOD2	CTGGACAAACCTCAGCCCTAAC	AACCTGAGCCTTGGACACCAAC
GAPDH	GTCTCCTCTGACTTCAACAGCG	ACCACCCTGTTGCTGTAGCCAA

Supplementary Table 2. Antibody list.

Antibody	Catalog number	Source
AIFM2/FSP1 mAb	#24972	Cellsignaling
Anti-IREB2 mAb	ab232994	Abcam
Anti-FBXL5 mAb	ab140175	Abcam
GPX4 mAb	#52455	Cellsignaling
ACSL4 mAb (F-4)	sc-365230	Santa Cruz
SOD2 (D9V9C) mAb	#13194	Cellsignaling
OCT 4 mAb	ab137427	Abcam
Snail mAb	ab216347	Abcam
Twist mAb	ab50887	Abcam
β-Actin mAb	#4967	Cellsignaling
